# Texture-Modified Diets, Nutritional Status and Mealtime Satisfaction: A Systematic Review

**DOI:** 10.3390/healthcare9060624

**Published:** 2021-05-24

**Authors:** Xiaojing Sharon Wu, Anna Miles, Andrea J. Braakhuis

**Affiliations:** 1 Faculty of Medical and Health Sciences, Discipline of Nutrition, The University of Auckland, Auckland 1010, New Zealand; a.braakhuis@auckland.ac.nz; 2 Faculty of Science, School of Psychology, Speech Science, The University of Auckland, Auckland 1010, New Zealand; a.miles@auckland.ac.nz

**Keywords:** texture-modified diet, dysphagia, swallowing impairments, older adults, malnutrition, mealtime satisfaction

## Abstract

While the association between dysphagia and malnutrition is well established, there is a lack of clarity regarding the nutritional status and mealtime satisfaction of those consuming texture-modified diets (TMDs). This systematic review summarises and critically appraises the nutritional status and mealtime satisfaction of adults consuming TMDs. A systematic database search following PICO criteria was conducted using Cochrane Central (via Ovid), MEDLINE, CINAHL, EMBASE and Scopus. Nutritional status, mealtime satisfaction and costs were identified as primary outcomes. Eligible studies were grouped according to outcome measurement. In total, 26 studies met the inclusion criteria. Twenty studies evaluated the nutritional status by weight change or using malnutrition screening tools and found the consumption of TMDs correlated with weight loss or malnutrition. Nine studies evaluated mealtime satisfaction, with two reporting poor satisfaction for people on thickened fluids (TFs). Nutrition intervention through adjusting texture and consistency and nutrition enrichment showed positive effects on weight and mealtime satisfaction. The majority of the studies were rated as ‘neutral’ quality due to the limited number of experiments. TMD consumers had compromised nutritional status and poor mealtime satisfaction. More research input is required to identify promising strategies for improving the nutritional status and mealtime satisfaction of this population. Food services need to consider texture, consistency and fortification in designing menus for people on TMDs to avoid weight loss and malnutrition, and to enhance mealtime enjoyment.

## 1. Introduction

Dysphagia has been defined as a difficulty in swallowing food or drink safely and efficiently, and can occur anywhere between the oral cavity to the stomach [1]. Approximately 8% of the global population are reported to suffer from dysphagia, and in the developed world, the prevalence appears to be increasing alongside the ageing population [2]. Neurological disease is a common cause of dysphagia, including acute stroke, Parkinson’s disease and dementia. Dysphagia can lead to severe consequences, including choking, aspiration, pneumonia and increased risk of mortality [1,3].

A high prevalence of weight loss and malnutrition has been reported in people with dysphagia, accompanied by nutrient deficiencies [4,5,6]. The co-existence of dysphagia and malnutrition has been reported in acute and long-term care (LTC) settings, with a higher prevalence of malnutrition for those with dysphagia [7,8,9]. Supporting this link, previous research has identified dysphagia as a decisive risk factor for malnutrition, and conducting nutritional and clinical interventions appears to reduce the incidence of malnutrition among dysphagic populations [10,11,12,13].

An essential and effective current clinical nutrition intervention for those with dysphagia is diet modification using prescribed texture-modified diets (TMDs), including thickened fluids (TFs) [3,11,14]. Standard TMDs are commonly softened, chopped, and minced or blended into the recommended texture and consistency by adding water.

Patients consuming TMDs may have negative perceptions of their meal experience. Poor compliance with dietary recommendations has been reported [15,16,17]. Possible causes include reduced mealtime pleasure from altered and restricted consistencies, prolonged mealtimes, swallowing exhaustion, physical difficulties in tolerating large fluid and food volumes, dependency of feeding, and associated cognitive impairments [18,19,20]. In addition, it is recognised that TMDs can be inherently less nutrient-dense due to the additional liquid requirements for processing [10]. This leads to an increased risk of compromised nutritional status [21,22,23].

Interventions have begun to be explored to optimise nutritional intake in order to lower the risk of unintentional weight loss and malnutrition. Some published reviews have indicated that nutritional supplements, meal fortification with nutrient-dense ingredients, energy-dense meals and offering nutritious in-between meals are considered as favourable interventions for malnutrition [24,25]. Other interventions include modifying meal contents or aesthetics by adjusting flavour, texture and consistency, and reforming the food appearance.

The association between TMDs, mealtime satisfaction and malnutrition warrants consideration by foodservice providers and carers who need to provide safe, nutritious and aesthetically appealing food to at-risk populations. The provision of food plays an essential role in health outcomes and quality of life for older adults [3]. Even in the context of a single meal, the ability to improve meal consumption and enjoyment exist. For example, offering variety within a meal increases consumption [26]. Several reviews have addressed TMDs in relation to (1) the swallowing safety of those with dysphagia [27,28,29], (2) the use of TMDs in specific populations [18,30], (3) quality of life [31] and (4) prevalence of dysphagia [5]. Therefore, while the safety and prevalence aspects of dysphagia have received research attention, the nutritional implications and mealtime satisfaction of TMDs have not been so thoroughly summarised and analysed. In a previous systematic review, we investigated nutritional intake in those on texture-modified diets [32]. The purpose of this paper was to systematically identify and examine the available evidence on nutritional status and mealtime satisfaction of adults using TMDs and TFs. The research questions were: what is the nutritional status of TMD consumers, and are they satisfied with the current TMDs?

## 2. Materials and Methods

This systematic review followed the PRISMA-P reporting checklist. The protocol was registered on PROSPERO CRD42019134897.

### 2.1. Selection Criteria

The PICO framework was used to assess study eligibility. Eligible study participants were prescribed any level of TMDs or TFs, and aged 18 years old. The adult age bracket was chosen to maximise data collection and study inclusion. Studies were excluded if participants were on a clear fluid diet, which is commonly prescribed to patients with gastrointestinal disease to minimise digestion rather than being an intervention diet for swallowing difficulties [33]. Studies were considered eligible if they assessed any of the following clinical measurements: anthropometry comparison or changes, nutritional status referring to the incidence of malnutrition, mealtime satisfaction and financial meal cost. Studies that reported the prevalence of malnutrition but did not include assessment details were excluded. Mealtime satisfaction, including commentary and/or ratings of TMDs or TFs, could be reported by either patients or staff. Experimental, observational, cohort and cross-sectional designs were included. Studies were excluded if they were case studies, reviews, expert opinions, conference paper, uncompleted clinical trials or non-English publications.

### 2.2. Data Sources

This systematic review was conducted alongside our previous review assessing the nutrition intake and meal content of TMDs in May 2019 [32]. The authors used the same database (Cochrane Central (via Ovid), MEDLINE, EMBASE, SCOPUS and CINAHL), and searching strategies, and updated the search in April 2021 (see Supplementary Data S1 for search strategy). With assistance from a senior librarian, search strategies were developed based on the various descriptions of TMDs and TFs.

### 2.3. Data Collection and Analysis

All search results were imported to Mendeley Desktop 1.19.4 (Mendeley, London, UK) [34]. One author screened titles and abstracts following PICO criteria. If the author considered the study eligible, a full-text article was retrieved and reviewed by two authors for final inclusion. The reference lists of full-text articles were manually screened for additional relevant studies. One author performed data extraction, crosschecked by the second author using a structured data collection form developed in Microsoft Excel for Office 365, version 1902 (Microsoft Corporation, Albuquerque, NM, USA) [35]. Data extracted included authors, year, settings, country of origin, study design, sample size, mean age, aetiologies, TMD/TF levels, interventions, nutritional status and mealtime satisfaction outcomes and measurement tools, key findings and limitations.

Eligible studies were categorised and summarised based on the outcome measurement (weight, nutrition status, mealtime satisfaction and cost). Observational studies and experimental studies were analysed individually under each outcome. Where three or more studies described the same intervention and outcome measurements, a meta-analysis was conducted.

The Academy of Nutrition and Dietetics Quality Criteria Checklist (QCC) for primary research was used for study quality assessment, including the risk of bias. Studies were graded as positive if issues of criteria, bias, generalisability, data collection and analysis were clearly addressed. Studies that were neither exceptionally strong nor exceptionally weak was graded as neutral. Negative studies indicate that not all issues have been adequately addressed.

## 3. Results

Following the PICO criteria, 26 eligible studies were included for final analysis, including thirteen observational studies, nine experimental studies and four RCTs. Title and abstract screening were conducted for 5239 non-duplicated studies. Fifty-eight articles were then chosen for full-text assessment, with an additional nine studies manually identified from reference lists (see Supplementary Data S2 for PRISMA chart). Two types of intervention were used to improve TMD quality. Firstly, to improve food appearance, shaping, moulding, texture/consistency modification were applied. Secondly, optimisation of the nutritional content was achieved through fortification or adding extra supplements.

### 3.1. Nutritional Status

Participant characteristics and study outcomes are presented in Table 1. Studies were conducted across 14 countries and various settings including hospitals (*n* = 11), LTCs (long-term cares) (*n* = 11), a combination of both (*n* = 2) or within the community (*n* = 2). Of the 26 studies, 19 were published after 2010. All 11 studies that assessed nutritional status used the MNA (Mini Nutrition Assessment) or MNA-SF (Mini Nutrition Assessment-Short Form) and were published in the last ten years. The age of the 7428 participants (1165 and 5863 from experimental and observational studies, respectively) ranged from 59 to 105 years old.

Though pureed food was the most common TMD studied, terminologies and descriptions of TMDs varied across countries. Types of TMDs/TFs included International Dysphagia Diet Standardisation Initiative (IDDSI) levels, UK descriptors (texture B, C, D, E), Japanese seven stages of TMDs, liquidized, mashed, minced, minced/pureed, chopped, soft and bite-sized, easy mastication and blended. TFs were labelled as honey-, nectar-, pudding-like, mildly thick and moderately thick viscosity, and Stage 1–3 from UK descriptors.

A total of 20 studies included measurements of body weight (BW), Body Mass Index (BMI), handgrip strength or malnutrition screening. Eleven observational studies assessed the nutritional status between TMD and standard diet consumers, as shown in Table 2.

A correlation between malnutrition or significant weight loss and consumption of traditional TMDs was found in all studies, with two exceptions [17,36]. On the other hand, using modified TMDs as an intervention showed positive weight changes compared to the traditional TMDs, found in all experimental studies (Table 3). Reyes-Torres et al. found handgrip strength was also significantly improved by consuming 12 weeks of consistency-modified TMDs [37]. The prevalence of malnutrition in patients consuming traditional TMDs ranged from 18.4% to 59%. MNA scores less than 17 or MNA-SF score less than 8 were classified as indicating malnutrition [38,39,40]. Zanini et al. reported the indicator of risk of malnutrition significantly reduced after 6 months of personalised textured TMDs (*p* < 0.001) despite scores still being in the range of at risk for malnutrition (MNA-SF = 10) [41]. Similar results were also found by Martín et al., with significant improvement of the malnutrition indicator (MNA-SF = 9.84 ± 2.05 vs. 11.31 ± 2.21, *p* = 0.0038) and a lower proportion of patients who were malnourished or at risk of malnutrition (78% vs. 34%, *p* = 0.0013) [42]. On hospital admission, dementia patients with dysphagia had a higher prevalence of malnutrition and a significantly higher risk of malnutrition compared to those without dysphagia [43]. Mean MNA-SF scores measured by Miles et al. and Vucea et al. also indicated the TMD consumers in LTCs were at risk of malnutrition [44,45]. Similar MNA-SF results were found in hospital patients consuming TMDs [39,46]. Additionally, hospitals offering multiple levels of TMDs (≥6 stages of TMDs) achieved improvements in both nutritional status and swallowing abilities [39].

**Table 1:** Characteristics of included studies in the systematic review.

Source	Method	Setting, Origin	Participant Characteristics	Interventions/ Intervention Period	Control	Outcomes	Quality Assessment *
Bannerman and McDermott (2011) [15]	Observational Cross-sectional	3 LTCs *Scotland*	Residents >60 y Ex: Nil by mouth, receiving artificial nutritional support, fluid restriction, acutely unwell, palliative Mean age (y) 88.1 ± 5.4	Texture C—Thicker pureed: *n* = 11 Texture D—Minced/moist: *n* = 4 [UK national descriptors 2009]	Standard diet *n* = 15	-Weight comparison -Nutritional status	Neutral
Cassen et al. (1996) [47]	Pre-post Experimental 16 days	LTC *US*	All residents consumed pureed diet Ex: Discharged or passed away	3D shaped pureed diet *n* = 18	Unshaped pureed diet *n* = 18	-Mealtime satisfaction (survey and staff report) -Cost	Neutral
Cassen et al. (1996) [47] *Follow-up study*	Cross-over cohort 12 m	LTC *US*	Residents consumed pureed diet for ≥1 m	6 m of 3D shaped pureed diet *n* = 13	Unshaped pureed diet *n* = 24	-Weight change	Neutral
Espinosa-Val et al. (2020) [43]	Prospective quasi-experimental	Hospital *Spain*	Dementia patients >18 y discharged from hospital Mean age (y) 84.1 ± 7.8	18 m follow up with recommendation and advice provided to family/caregivers	On admission *n* = 219 Standard *n* = 1 Easy mastication diet *n* = 117 Blended diet *n* = 88 Mixed diet *n* = 13	-Nutritional status	Neutral
Farrer et al. (2016) [48]	Pre-post Experimental 2 weeks	Hospital, *Australia*	Patients >18 y consuming pureed diet, medically stable and able to communicate	Moulded pureed diet (Texture C) *n* = 7	Unmoulded pureed diet (Texture C) *n* = 13	-Mealtime satisfaction (survey)	Neutral
Garon et al. (1997) [49]	RCT 1 year	Hospital stroke rehabilitation *UK*	Stroke patients with previously identified thin fluid aspiration by videofluoroscopy Mean age (y) 76.8	TFs + free access of water *n* = 10	TFs only *n* = 10	-Mealtime satisfaction (survey)	Positive
Gellrich et al. (2015) [50]	Observational Retrospective	38 clinics *Germany/Austria/Switzerland*	Patients with oral cancer *n* = 1526	Liquid, mashed	Standard diet	-Weight change	Neutral
Germain et al. (2006) [51]	RCT 12 weeks	LTC *Canada*	Residents aged 65–90 y admitted ≥3 m and had >7.5% weight loss in the last 3 m or BMI < 24 with dysphagia evaluated by RIC tool (Alzheimer’s *n* = 8, dementia *n* = 6, stroke *n* = 2, Parkinson’s *n* = 1) Ex. Cancer, chronic intestinal disease, terminally ill patients Mean age (y) 59	Shaped minced, minced/pureed or pureed diet and consistency-controlled TFs using Bostwick consistometer (nectar, honey, pudding) *n* = 9	Unshaped minced-70, minced-3 or pureed diet and uncontrolled honey-level TF (consistency not systematically controlled) *n* = 8	-Weight change	Neutral
Higashiguchi (2013) [52]	Experimental Cohort 7 days	17 hospitals/LTCs *Japan*	Inpatient and residents on TMDs with inadequate consumption (stroke *n* = 19, cancer *n* = 9, heart failure *n* = 7, fracture *n* = 5, dehydration *n* = 4, pressure ulcers, *n* = 3, pneumonia *n* = 2, anaemia *n* = 2, COPD *n* = 2, dementia *n* = 2, diabetes *n* = 1, Parkinson’s *n* = 1, other *n* = 17, none *n* = 2) (require total meal assistance *n* = 17, partial *n* = 6, none = 34) Mean age (y) 81.6 ± 9.3	3 days of nutrient-dense (enzyme-infused) TMDs nutrients were not diluted, and volume not increased *n* = 57	4 days of unmodified TMDs	-Mealtime satisfaction (Survey)	Positive
Karagiannis et al. (2011) [23]	RCT 8 days	Hospital subacute units *Australia*	Patients ≥18 y aspirated on thin liquids with prescription of modified or TF diet by SLTs without chronic respiratory conditions or prior tracheostomy Mean age (y) 79.5	TMDs (puree; minced; soft/minced) + TF (honey; pudding; nectar) + free access of water *n* = 13	TMDs + TF *n* = 5	-Mealtime satisfaction (survey)	Positive
Keller et al. (2012) [53]	Pre-post Experimental 9 m	Hospital and LTC *Canada*	All dysphagic residents fully consumed pureed or minced diets (stroke, Parkinson’s, dementia) Ex. Enteral feed Facility mean age 67 and 82 y	6 m of mix of 61% bulk and 39% shaped ready-to-use (reduced nutrients dilution and easier to chew and swallow) commercial TMDs *n* = 42	3 m of bulk commercial TMDs (unshaped, packaged in bulk)	-Weight change	Positive
Keller and Duizer (2014) [54]	Observational Interview	5 Rehabilitation and LTCs *Canada*	Consumed pureed diet for ≥1 week (stroke *n* = 6, delirium *n* = 2, spinal cord injury *n* = 1, diabetic coma *n* = 1, neck cancer *n* = 1, Parkinson’s *n* = 1, difficulty chewing/swallowing *n* = 3 Mean age (y) 77.3	None	Commercial (and in-house made) pureed diet *n* = 15	-Mealtime satisfaction (interview)	Neutral
Kennewell and Kokkinakos (2007) [55]	Observational Cross-sectional	2 hospitals *Australia*	Dysphagic patients	Infant-cereal fortified minced/pureed diets *n* = 17	Unfortified pureed diets	-Mealtime satisfaction (interview) -Cost	Neutral
Konishi and Kakimoto (2020) [56]	Observational Cross-sectional	LTC *Japan*	Older dementia residents had been admitted to a LTC between 2016–2019 Mean age (y) 87.9	Soft, *n* = 34 Chopped, *n* = 28 Blended, *n* = 9	Standard diet, *n* = 52	-Weight	Neutral
Maeda et al. (2019) [46]	Retrospective Observational	Hospital *Japan*	≥65 y admitted to an academic hospital during 2017–2018 with complete nutritional screening Mean age (y) 75.9 ± 7.0	TMDs, *n* = 110	Standard, *n* = 3484	-Weight -Nutritional status	Neutral
Massoulard et al. (2011) [17]	Observational Cross-sectional	4 LTCs *France*	All residents with chewing or swallowing difficulties Mean age (y) 85.8 ± 9.3	Chopped, *n* = 12 Mixed, *n* = 26	Standard diet, *n* = 49	-Weight comparison -Nutritional status	Neutral
Martín et al. (2018) [42]	Quasi-experimental 6 months	Hospital *Spain*	Acute geriatric unit patients ≥70 y diagnosed with OD during hospitalisation by nurses using volume-viscosity swallow test Mean age (y) 84.6 ± 5.5	14-day menus of TMDs (texture E or C) with TFs (nectar or pudding); ONS for malnourished or patients at risk of malnutrition; oral health recommendations *n* = 62	Standard hospitalisation recommendations, which were not applied systematically nor in a standardised individualised application *n* = 124	-Weight change -Nutritional status	Positive
Miles et al. (2019) [44]	Observational Cross-sectional	12 LTCs *New Zealand*	Residents consuming > 3 servings/day of commercial TMDs (dementia *n* = 37, cognitive impairment *n* = 65, brain injury *n* = 25, progressive neurological disease *n* = 9) Mean age (y) 85 ± 7.7	None	Commercial fortified TMDs and TFs *n* = 67	-Weight comparison -Nutritional status -Mealtime satisfaction (interview)	Neutral
Okabe et al. (2016) [57]	Observational Cohort with 1-year follow up	2 mid-sized cities *Japan*	≥60 y living at home or using in-home nursing care without malnutrition	None	Minced/pureed/mixed *n* = 339	-Nutritional status	Neutral
Ott et al. (2019) [58]	Pre-post Experimental 12 weeks	2 LTCs, *Germany*	Residents diagnosed with chewing or swallowing receiving TMDs regularly (all participants had cognition impairment) Mean age (y) 86.5 ± 7.4	6 weeks of usual TMDs (completely pureed or partial soft food) *n* = 16	6 weeks of one level of reshaped TMDs and enriched with 600 kcal energy and 30 g protein *n* = 16	-Weight change -Mealtime satisfaction (interview)	Neutral
Reyes-Torres et al. (2019) [37]	RCT 12 weeks	National Institute, *Brazil*	≥65 y with a caregiver and a confirmed diagnosis of oropharyngeal dysphagia, and consumed TMDs and TFs (evaluated by V-VST and EAT by dietitians) Mean age (y) 76	Consistency-modified and standardised TMDs and nectar or pudding level TFs (measured with Brookfield viscometer) *n* = 20	Unmodified pureed diet with one viscosity of TFs (consistency not systematically controlled) *n* = 20	-Weight change -Handgrip -Nutritional status	Positive
Shimizu et al. (2018) [59]	Retrospective Cross-sectional	Hospital rehabilitation ward, *Japan*	≥65 y patients Ex. Tube feeding, parenteral nutrition, history of stroke, neurodegenerative disease Mean age (y) 80.6 ± 7.5	TMDs, *n* = 22	Standard diet, *n* = 123	-Weight -Nutritional status	
Shimizu et al. (2020) [39]	Retrospective Cohort	7 rehabilitation facilities, *Japan*	≥65 y with pneumonia enrolled in rehabilitation facilities with record of malnutrition screening at admission and discharge Mean age (y) 82.9 ± 9.8	Providing multiple TMD stage ≥ 6 *n* = 109	Providing TMD stage < 6 *n* = 109	-Weight -Nutritional status change	Neutral
Vucea et al. (2019) [45]	Observational Cross-sectional	32 LTCs, *Canada*	Randomly recruited residents > 65 y admitted for ≥1 m Mean age (y) 86.8 ± 7.8	TMDs Bite-sized, *n* = 91 Minced, *n* = 139 Pureed, *n* = 68	Standard diet, *n* = 338	-Nutritional status	Positive
Welch et al. (1991) [60]	Pre-post Experimental 6 m	LTC *US*	Residents consumed pureed diet and weighed below average or serum albumin/transferrin levels below normal values (identified from medical records) Mean age (y) 81	Pureed diets with fortified high-fibre cereals and commercial supplements *n* = 15	Pureed diets with unfortified cereals	-Weight change	Neutral
Wright et al. (2005) [36]	Observational Cross-sectional	Hospital elderly and neurology wards *UK*	All medically stable patients consumed TMDs or standard diet (reasons for TMDs: 80% dysphagia, 20% poor dental state; stroke *n* = 19, fall *n* = 8, other *n* = 3) Mean age (y) 81.5	Texture B—Smooth pureed, *n* = 10 Texture D—Minced/mashed, *n* = 9 Texture E—Soft, *n* = 11 (UK national descriptors, 2002)	Standard diet, *n* = 25	-Weight comparison	Neutral
Zanini et al. (2017) [41]	Pre-post experimental 6 m	20 LTCs Italy	Dysphagic residents >65 y with low comorbidity levels (diagnosed by a physician or reported in medical records) Mean age (y) 79.72 ± 12.31	6 m of personal-modified levels of density, viscosity, texture and particle size TMDs *n* = 401	6 m of unmodified TMDs	-Weight change -Nutritional status -Mealtime satisfaction (EdFED)	Positive

Note. RCT—randomized control trial; Ex.—exclusions; BMI—body mass index; LTC—long-term care; y—years old; TMD—texture-modified diet; TF—thickened fluids; RIC tool—Rehabilitation Institute of Chicago Clinical Evaluation Dysphagia; SLT—speech-language therapist; ONS—oral nutrition supplement. m—months. * Quality of the study was assessed using Quality Criteria Checklist (QCC). Positive studies were identified as clearly addressed issues of criteria, bias, generalisability, data collection and analysis. Neutral studies were indicated as neither exceptionally strong nor exceptionally week.

**Table 2:** Outcome data for observational studies assessing nutritional status.

Studies	BMI/Weight Outcomes	MNA-SF Outcomes	Nutritional Status Findings
Bannerman and McDermott [61]	TMDs vs. Std 18.4 ± 2.6 vs. 22.1 ± 2.8, *p* = 0.001		TMDs vs. Std % Underweight (BMI < 18.5): 60.0% vs. 6.7% (*n* = 9 vs. 1)
Konishi and Kakimoto [56]	TMDs vs. Std Mild dementia: 19.4 vs. 22.2, *p* = 0.0686 Severe dementia: 19.4 vs. 21.5, *p* = 0.0077		
Gellrich et al. [50]	Oral cancer patients on liquid (61%) and mashed (51%) diets were more likely to lose weight 46% on Std were able to maintain weight. Patients who had >10 kg weight loss more frequently had to eat mashed food compared to those who had ≤10 kg weight loss were more frequently able to eat Std (*p* < 0.001).		
Maeda et al. [46]	TMDs vs. Std 19.0 ± 3.8 vs. 22.4 ± 3.5, *p* < 0.001	TMDs vs. Std 6.8 ± 2.5 vs. 11.6 ± 2.2, *p* < 0.001	TMDs vs. Std % Malnourished: 62.7% vs. 6.1% (*n* = 69 vs. 213) % At risk of malnutrition: 36.4% vs. 35.3% (*n* = 40 vs. 1231) % Well-nourished: 0.9% vs. 58.6% (*n* = 1 vs. 2040)
Massoulard et al. [17] ^(a)^			TMDs vs. Std % Malnourished: 18.4% vs. 30.6% (*n* = 7/38 vs. 15/49), *p* = 0.3 % Obesity: 31.2% vs. 38.8% (*n* = 12/38 vs. 19/49)
Miles et al. [44]	LTC residents consuming fortified TMDs Mean BMI: 23 ± 5.22 (13–35) ↑ number of weeks on fortified TMDs was sig. associated with ↑ age (*p* < 0.05), ↓ BMI or weight (*p* < 0.05) and ↑ supplement use (*p* < 0.001)	Fortified TMDs Mean MNA-SF = 8 ↓ MNA-SF scores were sig. correlated with ↓ BMI (*p* < 0.05) and more medical conditions (*p* < 0.05)	Fortified TMDs % Underweight (BMI < 18.5): 22.0% (*n* = 9/41) % Overweight (BMI ≥ 25): 29.3% (*n* = 12/41) % Malnourished: 35.5% (*n* = 11/31) % At risk of malnutrition: 61.3% (*n* = 19/31)
Okabe et al. [57]			50.0% (*n* = 8/16) of malnourished participant were on TMDs (MNA-SF) 71.4% (*n* = 5/7) of participants who passed away were on TMDs Consumption of TMDs (RR: 2.93, *p* = 0.036) and swallowing disorder (RR: 3.82, *p* = 0.012) was sig. associated with the incidence of malnutrition and death among frail older adults at 1-year follow-up.
Shimizu et al. (2018) [59]	TMDs vs. Std 19.1 ± 3.4 vs. 20.3 ± 3.5, *p* < 0.001		TMDs vs. Std % Malnourished: 59.1% vs. 35.8% (*n* = 13 vs. 44) % At risk of malnutrition: 40.9% vs. 52.8% (*n* = 9 vs. 65) % Well-nourished: 0% vs. 11.4% (*n* = 0 vs. 14)
Shimizu et al. [39]	Comparison between multiple TMDs (*n* = 109) and control (<6 stages of TMDs) patients (*n* = 109) At admission: MNA-SF: 8 (6–10) vs. 8 (6–10), *p* = 0.969; BMI: 20.1 ± 4.4 vs. 19.8 ± 4.4, *p* = 0.486 At discharge: MNA-SF: 6 (4–8) vs. 6 (3–8), *p* = 0.459 MNA-SF change during hospitalisation: 2.4 ± 2.8 vs. 0.9 ± 3.1 (*p* < 0.001)		
Vucea et al. [45]	TMDs vs. Std 23.97 ± 5.24 vs. 26.57 ± 5.92, *p* < 0.001	TMD vs. Std 9.81 ± 2.68 vs. 11.37 ± 2.13, *p* < 0.01	BMI and MNA-SF score the lowest in pureed < minced and moist < soft and bite-sized < Std. MNA-SF was sig. negative (↑ risk of malnutrition) associated with minced and moist and pureed diet compared to Std (*p* = 0.03)
Wright et al. [36]	TMDs vs. Std: 60 (39–96) vs. 62 (46–93) kg, *p* = 0.55		

Note. BMI—body mass index; TMD—texture-modified diet; Std—standard diet; Sig—significant; ↓—low; ↑—increased; MNA-SF scores: 0–7 = malnourished; 8–11 = at risk of malnutrition; 12–14 = normal nutritional status/well-nourished. ^(a)^ Malnutrition—BMI < 21 OR ≥ 10% weight loss in 6 months/5% in 1 m month OR MNA < 17 (used when MNA-SF < 11); normal BMI = 21–29.9.

**Table 3:** Outcome data for experimental studies assessing nutritional status.

Studies	BMI/Weight Outcomes	MNA-SF Outcomes	Nutritional Status Findings
Cassen et al. [47]	3D moulded vs. unmoulded TMDs NS weight loss in 6 months: 15.4% vs. 100% (*n* = 2/13 vs. 21/21) Sig. weight loss of ≥4.5 kg 0% 19% (*n* = 0/13 vs. 4/21)		
Espinosa-Val et al. [43]		Dysphagia vs. Non-dysphagia patients: 7 ± 2.68 (*n* = 211) vs. 8.2 ± 2.45 (*n* = 35), *p* = 0.014	% Malnourished: 53.6% (*n* = 113) % At risk of malnutrition: 43.1% (*n* = 91) % Well-nourished: 3.3% (*n* = 7)
Germain et al. [51]	Shaped vs. unshaped TMDs 6 weeks: NS weight change (*p* > 0.05) 12 weeks: Sig. ↑ weight in shaped TMDs and weight loss was seen in unshaped TMDs +3.90 ± 2.3 0 vs. −0.79 ± 4.18 kg, *p* < 0.05 BMI ↑ from 22.4 ± 3.93→24.5 ± 4.14		
Keller et al. [53]	74% of participants consuming mix of shaped ready-to-use TMDs and bulk TMDs achieved weight goal after 6 months NS weight or morbidity change between intervention and control periods Trends towards ↑ weight on mixed TMDs (OR = 3.5, *p* = 0.16) and ↓ weight on bulk TMDs (OR = 4.3, *p* = 0.11)		
Martín et al. [42]	Standardised vs. non-standardised TMDs BMI within normal range at both admission and 6-month follow up Changes in 6 months intervention 27.76 ± 4.42 vs. 28.52 ± 4.39, *p* = 0.2045	Changes in 6 months intervention 9.84 ± 2.05 vs. 11.31 ± 2.21, *p* = 0.0038	Changes in 6 months intervention % Malnourished: 18.75% vs. 3.13% (*n* = 6 vs. 1) % At risk of malnutrition: 59.38% vs. 31.25% (*n* = 19 vs. 10) % Well-nourished patients 21.87% vs. 65.63% (*n* = 7 vs. 17), *p* = 0.0013
Ott et al. [58]	Weight change during 6 weeks traditional TMDs: 59.3 vs. 58.8 kg (−0.5 kg), *p* = 0.21 Weight change during 6 weeks enriched and shaped TMDs: 59.6 vs. 58.8 (+0.8 kg) kg, *p* = 0.007		Baseline MNA-SF % Malnourished: 25% ((*n* = 4/16) % At risk of malnutrition: 75% (*n* = 12/16)
Reyes—Torres et al. [37]	Weight change after 12 weeks consistency modified TMDs: 56 ± 10 vs. 60 ± 10 (+7%) kg, *p* < 0.001 Handgrip strength:18 ± 11 vs. 21 ± 13 kg, *p* = 0.004 NS Weight/BMI changes in traditional TMDs control group (*p* > 0.05)		Baseline MNA % Malnourished: 50% (*n* = 20/40)
Welch et al. [60]	Weight change after 3 months vs. 6 months of fortified TMDs and supplements +2.8 ± 1.25 vs. +4.6 ± 2.0 lbs, *p* < 0.04 66.7% ↑ 0.5–5.4 kg from 3–6 month; 33.3% ↓ 0.5–2.3 kg		
Zanini et al. [41]	Changes after 6 months personalised TMDs 17.88 ± 3.48 to 19.00 ± 3.32(+1.12), *p* < 0.001 with sig. growth trend (*p* < 0.007) Changes after 6 months traditional TMDs control group 20.96 ± 4.07 vs. 17.88 ± 3.48 (−3.08), *p* < 0.001	Changes after 6 months personalised TMD 8 to 10 (+2), *p* < 0.001 Changes after 6 months traditional TMDs control group 7 to 8 (−1), *p* < 0.001	

Note. BMI—body mass index; TMD—texture-modified diet; Sig—significant; NS—no significant; ↓—low; ↑—increased. MNA-SF scores: 0–7 = malnourished; 8–11 = at risk of malnutrition; 12–14 = normal nutritional status. MNA scores: 0–16 = malnourished; 17–23.5 = at risk of malnutrition; 24–30 = well-nourished.

### 3.2. Mealtime Satisfaction

The use of an interview was the most common method for exploring mealtime satisfaction [44,54,55,58]. Three studies evaluated mealtime satisfaction for shaped/moulded TMDs by survey [5,47,48]. Keller and Duizer found that despite LTC consumers appreciating the necessity for TMDs and staff efforts, several issues contributed to the poor acceptance of pureed meals, including presentation, taste, smell, inconsistency in production, delivery and lack of variety [54].

Zanini et al. used the Edinburgh Feeding Evaluation in Dementia (EdFED) scale, which observed participants’ adverse behaviours while being fed [41]. A significant improvement in eating behaviour was found with adjusted TMDs by the texture-individualised intervention (6.65 ± 3.13 vs. 7.98 ± 3.65, *p* < 0.001), which was demonstrated by a high level of compliance to meal consumption [41]. Results from studies using moulded pureed foods were inconsistent. No significant differences were found in the 14-day hospital trial with moulded pureed food and 3-day trial of enzyme-infused shaped TMDs with regards to taste, appetite, presentation, ease of ingestion and overall liking (*p* > 0.05) [48,52]. Nevertheless, health professionals reported shaped TMDs to be significantly more appealing (4.70 vs. 4.22, *p* < 0.01), with a significantly higher score in joy of eating (4.44 vs. 4.11, *p* < 0.001) and overall satisfaction (4.38 vs. 4.19, *p* < 0.05) after observing patient consumption [52]. Positive feedback from staff and LTC residents was also obtained with a 16-day 3D pureed food trial [47].

Miles et al. reported that LTC residents were satisfied with the appearance and flavour of commercial fortified TMDs, with the highest satisfaction with puddings and soups (8–10 out of 10) [40]. Dryness of the meals was reported by hospital patients consuming infant cereal fortified TMDs [55].

Ott et al. reported mixed feedback from nursing staff regarding enriched and shaped TMDs [58]. Improvement of chewing or swallowing (*n* = 5/16), enhanced appetite and pleasure with eating was observed in some participants (*n* = 5/16), while one participant did not like the taste. Concerningly, 33% of the residents (*n* = 5/15) were also rated by nursing staff as not receiving their prescribed diet consistency [58].

Garon et al. and Karagiannis et al. evaluated mealtime satisfaction of TFs and the alternative intervention of free water access using a survey [23,49]. Patients with access to water had a significantly higher level of satisfaction with drinks, level of thirst and mouth cleanliness (*p* < 0.001), but no significant differences in the overall feelings [23]. Garon et al. found similar results, with high satisfaction with access to water [49]. Moreover, 90% of patients on TFs (*n* = 9/10) reported a desire for water due to thirst, and that TFs were not thirst-quenching and lacked taste and enjoyment. Only one person in the study was satisfied with TFs.

### 3.3. Cost

The cost of TMD provision was only discussed in two studies [47,55]. Using infant cereal as fortification was 6.9 times cheaper compared to commercially fortified thickener in Australia ($0.235 vs. $1.61 AUD) [55]. Cassen et al. suggested that using enhancer and thickener in 3D moulded foods could be cost-effective in increasing food intake to prevent weight loss and reduce the use of supplements and treatments [47].

### 3.4. Quality Assessment

Overall, 65% (*n* = 17) of the studies were rated as ‘neutral’ quality due to the absence of follow-up. Despite 12 studies being experimental studies, only eight were rated as ‘positive’ quality. The other four experimental studies were rated as ‘neutral’ quality, mainly due to the small number of subjects and a lack of specification of drop-outs. Sample size largely varied, from 15 to 3594 [50,54]. All studies had evenly distributed numbers of participants between study groups. Results of nutritional status were collected using validated tools. Evaluation of mealtime satisfaction varied across studies depending on the questions and survey they used, as well as the cognition status of the study group. Meta-analysis was not possible due to the heterogeneity of study methods and settings and a small number of studies measuring the same outcomes.

## 4. Discussion

This systematic review evaluated the existing evidence of impact of TMDs and TFs on nutritional status and mealtime satisfaction. Compromised nutritional status was identified in TMD consumers. Modifying TMDs by shaping and nutrition enrichment showed promising results in improving nutritional status and mealtime satisfaction.

### 4.1. Measuring Nutritional Status

A previous literature review studied the prevalence of malnutrition in LTCs, indicating malnutrition risk is 1.7 times higher in LTC residents consuming TMDs compared with those on standard diets [5,62]. A finding mimicked in the current review. Malnutrition was reported in 33–50% of dysphagic residents in LTCs and approximately 60% in hospital [37,44,46,59].

Besides the high prevalence of malnutrition in dysphagia patients, compared to standard diet patients, those consuming TMDs were found to have poor long-term clinical outcomes, such as respiratory infection, decreased skeletal muscle mass, longer hospital stays and higher mortality rate [39,43,46,59]. However, the association between malnutrition, dysphagia and mortality does require further clarification. Ten studies used MNA-SF, and one used MNA for examining malnutrition. Although MNA-SF is validated for determining nutritional status in the LTC population, the inclusion of functional, psychological, and cognitive status may result in overestimation of malnutrition risk ratings. There was a lack of screening and assessment of dysphagia and malnutrition [63,64]. Only Zanini et al. and Martín et al. compared the malnutrition screening score before and after intervention and found improvement, whereas other studies only did the screening at baseline [41,42].

Weight loss was more significant in oral cancer patients on TMDs compared to those on standard diets in a retrospective study [50]. Missing documentation or measurement of weight and height was observed in LTCs, suggesting inadequate monitoring [44]. The risk of malnutrition is highly associated with weight and BMI, and as such regular assessment and documentation are necessary. A recent review suggested using the Global Leadership Initiative on Malnutrition (GLIM) criteria to assess the nutritional status of dysphagic adult patients. This covers BMI, nutritional screening tools, anthropometric measurements, body composition and dietary assessment [65]. Standardised nutritional assessment, nutrition recommendations for texture and nutrients should be included as part of dysphagia diagnosis and treatment plan [63,64,66].

### 4.2. Improving Nutritional Status

Martín et al. demonstrated that the introduction of TMDs in combination with the use of high-protein, high-energy foods and an oral health intervention could significantly improve both functional and nutritional status in hospital patients with dysphagia [42]. Of eight intervention studies, significant weight or BMI improvements were found in three modified TMDs intervention studies [37,41,51]. Inadequate dietary intake may be contributing to the high prevalence of undernutrition [15]. The impact of nutrient enrichment on weight was only investigated in one study that reported a significant weight improvement in a LTC using a pureed diet with supplements and fibre-fortified cereals [60]. Results from previous research studying standard diets suggest food fortification improves energy and protein intake, but not body composition or functional status in nursing home participants at nutritional risk [67]. Similar results were found by Miles and colleagues, with 97% of the participants on fortified TMDs at risk of malnutrition at the time of investigation [44]. However, as an observational study, it is not possible to determine whether the outcomes were coincidental. Though nutritional status was found to be routinely screened in nursing homes, diet quality is one of the biggest challenges of clinical management which can result in malnutrition [68,69]. The previous review summarised that the positive improvement in nutrition intake was found using fortification and shaped TMDs [32]. Implementing nutrition enrichment strategies such as fortified food or supplements should be considered in dysphagic management for malnourished patients and those who are at risk of malnutrition [68,70].

### 4.3. Mealtime Satisfaction

Improved energy and protein intake were achieved by optimising the texture modification [32,71]. The quality of TMDs is highly related to mealtime acceptance and compliance. Therefore, to guarantee adequate intake, studying mealtime satisfaction is crucial. While studies did focus on both the aesthetics and safety of the food, there was a lack of standardisation in the evaluation of mealtime satisfaction of TMDs and TFs. Poor satisfaction with TFs was found in hospital patients, resulting in poor compliance and consumption [23,49,72]. Moreover, 3D printing and shaping using silicone moulds have the potential to achieve more attractive TMDs with enhanced sensory characteristics and consistency [73]. Though no significant results were reported by patients directly, improvements were observed by staff and health professionals with the use of shaping/moulding [47,52]. Dysphagic patients require greater attention and encouragement on the acceptance of the texture modifications. Targeting additional feeding support to high-risk patients may improve nutritional status and outcome [74,75]. Clinical staff should collaborate with food service to improve meal presentation and aesthetics, as well as raising concerns regarding the appropriate texture and consistency necessary for safe swallowing. Responses using subjective measurement may be limited due to common cognitive challenges in this population. Studies may consider using observations or audits to assess mealtime satisfaction [23].

Ballesteros et al. suggested nutrient-dense commercial TMD product could be an inexpensive, safe and convenient alternative for dysphagic patients [76,77]. Our referenced studies support the use of fortification in TMDs as a cost-effective approach to improving nutritional intake [47,55]. Similar findings have been seen in studies of hospital patients on regular diets where the use of food fortification has improved nutritional intake [78,79]. Considering the increasing use of TMDs, future studies should include cost effectiveness of fortification, thickeners, shaping powder and moulds used for shaping/moulding as references for food service.

The participants included in the review had various conditions, and included acute patients and chronic aged-care residents. Existing medical conditions may affect the assessment of nutritional status and responses of mealtime satisfaction. Future studies should consider analysing the association between patient aetiologies, clinical reasons related to TMD consumptions, nutritional status and mealtime satisfaction. The results may not be generalisable to individual patients with specific conditions. Evaluation of other clinical signs related to nutritional status and biochemistry makers, such as bowel movement, wound healing and blood test of micronutrient level should be considered in future studies, as very few studies incorporated these.

## 5. Conclusions

Poor nutritional status and meal acceptance was found in TMD and TF consumers. Although nutrition enrichment through fortification and modifying the texture and consistency of TMDs through shaping, moulding and consistency-control are promising strategies for improving nutritional status and preventing weight loss, there was a lack of evidence to make firm conclusions. The majority of the evidence used in this review was rated as neither strong nor weak. More high-quality research with follow-up and different interventions is required to generalise for larger populations. Future interventions should collaborate with food service to implement high-quality, nutritious TMDs, focusing on improving the nutrition content, appearance, flavour, taste, varieties and consistency.

## Data Availability

The data used and/or analysed during the current study are available from the corresponding author on reasonable request.

## Supplementary Materials

The following are available online at https://www.mdpi.com/article/10.3390/healthcare9060624/s1, S1: Search strategy; S2: Prisma flow chart.
