# Supplementary material for: Texture-Modified Diets, Nutritional Status and Mealtime Satisfaction: A Systematic Review
Source: Healthcare (Basel). 2021 May 24;9(6):624. doi: 10.3390/healthcare9060624 (PMC8225071; doi:10.3390/healthcare9060624)
Supplement: Supplementary file 1 [file healthcare-09-00624-s001.zip › Supplementary data S1 Search strategy.pdf]

Supplementary data S1 Search strategy

COCHARANE via Central

1. Food OR diet OR meal OR nutrition in Title Abstract Keyword
2. modifi* OR modify* OR TMD OR puree* OR mince* OR blend* OR chop* OR soft OR thicken* OR liquid* OR solid* OR fluid* in Title Abstract Keyword
3. adequa* OR require* OR supplement* OR fortificat* OR snack* OR intake OR energy OR protein OR micronutri* OR satisfact* OR complian* OR quality* in Title Abstract Keyword
4. 1 and 2 and 3

MEDLINE (Ovid)

1. (texture* adj1 modif* adj (food or foods or diet or diets or meal or meals)).ti,ab,kw.
2. ((puree or pureed or mince* or blend* or chop* or soft or soften* or thicken* or liquid* or liquef*) adj1 (food or foods or diet or diets or meal or meals or fluid or fluids)).mp.
3. 1 or 2
4. limit 3 to "humans only (removes records about animals)"
5. limit 4 to ("young adult (19 to 24 years)" or "adult (19 to 44 years)" or "young adult and adult (19-24 and 19-44)" or "middle age (45 to 64 years)" or "middle aged (45 plus years)" or "all aged (65 and over)" or "aged (80 and over)")

EMBASE (Ovid)

1. (texture* adj1 modif* adj (food or foods or diet or diets or meal or meals)).ti,ab,kw.
2. ((puree or pureed or mince* or blend* or chop* or soft or soften* or thicken* or liquid* or liquef*) adj1 (food or foods or diet or diets or meal or meals or fluid or fluids)).mp.
3. 1 or 2
4. limit 3 to "humans only (removes records about animals)"
5. limit 4 to (adult <18 to 64 years> or aged <65+ years>)

SCOUPUS

1. TITLE-ABS-KEY ((texture* W/1 modif*) W/1 (food* or diet* or meal*))
2. TITLE-ABS-KEY ((puree* or mince* or blend* or chop* or soft or soften* OR thicken* or liquid* or liquef*) W/1 (food* or diet* or meal* or fluid*))
3. 1 and 2

CINAL PLUS (EBSCOhost)

1. texture* N1 modif* N1 (food* or diet* or meal*
2. ((puree* or mince* or blend* or chop* or soft or soften* or thicken* or liquid* or liquef*) N1 (food* or diet* or meal* or fluid*))
3. 1 or 2

Search Results:

Search date: 5th May 2019

CENTRAL - (Cochrane Central Register of Controlled Trials), 1450 results
MEDLINE (Ovid), 2313 results.
EMBASE (Ovid), 2887 results
SCOPUS, 51 results, 2 more result was found at 25th June 2019

CINAL Plus (EBSCOhost), 464 results

Updated search date: 1st April 2021

CENTRAL - (Cochrane Central Register of Controlled Trials), 88 results
MEDLINE (Ovid),161 results.
EMBASE (Ovid), 913 results
SCOPUS, 14 results
CINAL Plus (EBSCOhost), 78 results
