# Supplementary material for: Texture-Modified Diets, Nutritional Status and Mealtime Satisfaction: A Systematic Review
Source: Healthcare (Basel). 2021 May 24;9(6):624. doi: 10.3390/healthcare9060624 (PMC8225071; doi:10.3390/healthcare9060624)
Supplement: Supplementary file 1 [file healthcare-09-00624-s001.zip › Supplementary data S2 PRISMA FLOW CHART.pdf]

Supplementary data S2

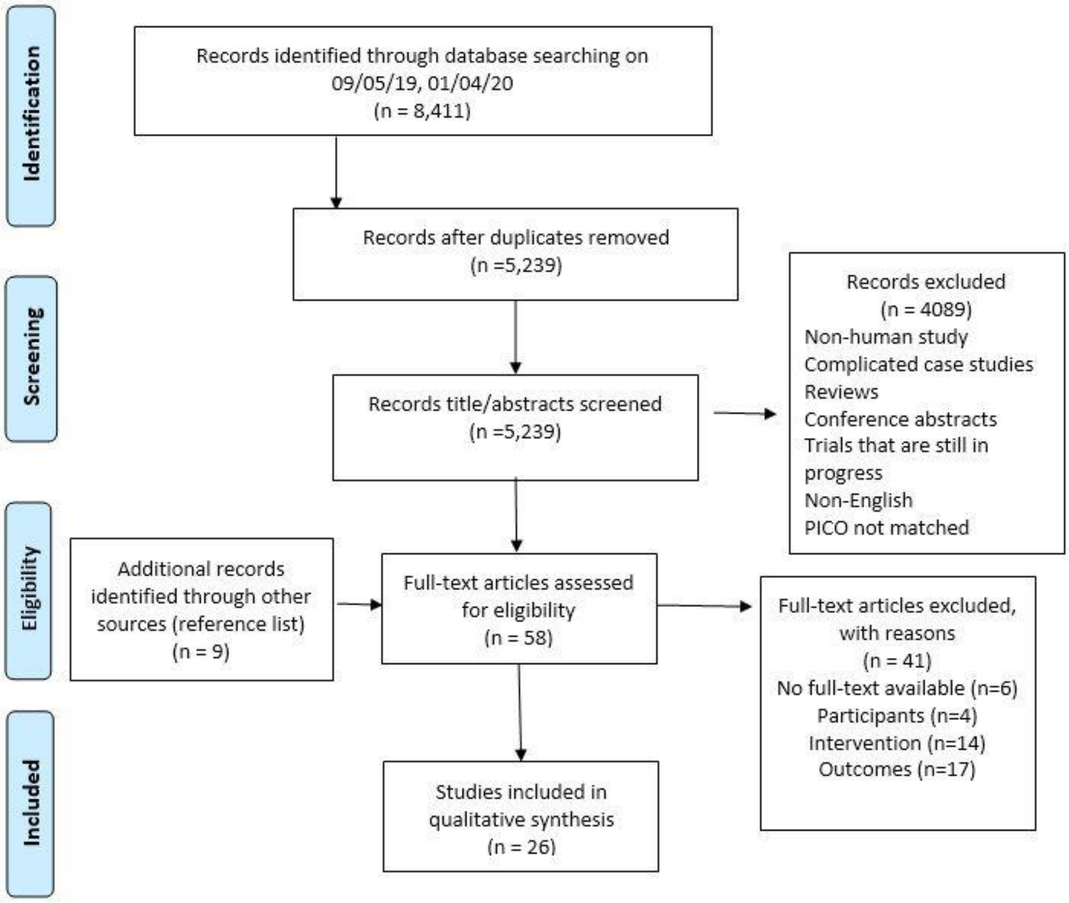

Figure 1. PRISMA flow chart diagram of study selection process.
Note. Lab studies without human participants (participants), studies without TMDs or TFs consumers (intervention) or study outcomes did not include nutrition measurement (outcomes) were excluded.
